# A Great Man Gone

**Published:** 1916-09

**Authors:** 


### A Great Man Gone.

On August 11th, Dr. John B. Murphy, distinguished surgeon and philanthropist, passed to his reward in the great beyond. Dr. Murphy was one of the noblest and best men that has ever honored the medical profession in this country, and his untimely death will be mourned by the whole profession.

